# Desmosomes and Desmosomal Cadherin Function in Skin and Heart Diseases—Advancements in Basic and Clinical Research

**DOI:** 10.1155/2010/725647

**Published:** 2010-09-22

**Authors:** Mỹ G. Mahoney, Eliane J. Müller, Peter J. Koch

**Affiliations:** ^1^Departments of Dermatology and Cutaneous Biology and Biochemistry and Molecular Biology, Thomas Jefferson University, Philadelphia, PA 19107, USA; ^2^Molecular Dermatology, Institute of Animal Pathology, University of Bern, 3001 Bern, Switzerland; ^3^Departments of Dermatology and Cell and Developmental Biology, Charles C Gates Center for Regenerative Medicine & Stem Cell Biology, University of Colorado Denver, Denver, CO 80217, USA

In the last two decades desmosomal research has developed from a highly specialized area of cell biology to an area of biomedical research aimed at elucidating the role of these cell junctions in tissue and organ development and homeostasis. 

Until the late 1980s, desmosomal research focused, to a large extent, on the morphological characterization of desmosomes and the biochemical identification of desmosomal proteins. Towards the end of that decade, the first desmosomal transmembrane proteins (desmogleins and desmocollins) were cloned, demonstrating that these proteins are sequence related to cadherins, a family of calcium-dependent cell adhesion proteins. Subsequent knockout experiments in mice, published in the 1990s, demonstrated that at least some of the desmosomal cadherins (e.g., desmoglein 3) are required to maintain tissue integrity in the oral mucosa (see the paper of Ganeshan et al.). Around the same time, it was shown that patients who suffer from autoimmune diseases characterized by skin and mucous membrane blistering produce autoantibodies against desmogleins 1 and 3 (DSG1, DSG3). These two observations suggested that the loss of normal desmosome function could lead to tissue fragility disorders.

Subsequent to the identification of pemphigus as a desmosomal disease, reports began to emerge suggesting that mutations in desmosomal genes can cause a variety of skin blistering disorders and cardiomyopathy (see the paper by J. Li and G. L. Radice).

Recently the question has emerged: Are all desmosomal diseases caused by a loss of cell adhesion? Mounting evidence suggests that abnormal cell signaling might contribute to the pathophysiology of at least certain types of desmosomal diseases, such as pemphigus (see the paper of M. Bektas et al. and D. Brennan et al.). The putative contributions of cell adhesion defects and abnormal cell signaling in diseases like pemphigus are still under intense debate. We can expect to see a continued influx of exciting new findings in this area.

The present special issue is a collection of reviews and original research articles that focus on fundamental aspects of desmosome biology and on clinical research relating to desmosomal diseases of the skin, mucous membranes, and heart. These papers are also examples of how a synergistic approach that utilizes the tools of genetic engineering in mice, human genetics, cell and molecular biology and immunology have led to a significant advancement of our understanding of the pathological mechanisms underlying desmosomal diseases. We sincerely thank the authors for their high-quality and exciting contributions. We hope that these provocative papers will stimulate discussions and promote future collaborations.

##  Basic Biological Features

The paper entitled “*Desmosomes in vivo*” D. Garrod discusses the importance of desmosome assembly and disassembly during development and in disorders such as blistering diseases of the skin. Most of our understanding of the underlying regulatory mechanisms has been gathered from investigations on cultured cells where desmosomes are in a calcium-dependent as opposed to a calcium-independent or hyperadhesive state. This thought-provoking review from a leader in the desmosomal field critically discusses the state of desmosomes in cell culture and in vivo, the properties of calcium-independent desmosomes, and their reversion to a calcium-dependent state during wound healing. Highly instructive for researchers interested in disorders affecting desmosomal structure and function, this paper is a “must” for young investigators in the field (Editor: E. J. Müller).

The paper entitled “*Exploring the nature of desmosomal cadherin associations in 3D*”, by G. R. Owen and D. L. Stokes, states that the expression of desmosomal proteins is highly regulated in both a spatial and temporal manner, thus establishing an intricate, dynamic network of proteins that must provide both structural stability as well as flexibility to cells and tissues. This paper highlights the use of a state-of-the-art technique known as high-resolution electron tomography, to examine the interactions between cadherins within the desmosome structure. The findings from these studies offer us an architectural perspective of cadherin interactions and how the binding of pathogenic pemphigus antibodies to these cadherins could disrupt desmosome adhesion and induce intra-epithelial blistering (Editor: M. G. Mahoney).

“*Leaving the desmosome—the desmosomal plaque proteins of the plakophilin family*” by S. Neuber et al. reviews the diverse cell adhesion-dependent and adhesion-independent functions of junctional proteins. In this paper, the authors discuss the plakophilin family of proteins and the complexity of their roles, which ranges from cell signaling to organization of the cytoskeleton and control of protein biosynthesis. Aberrant expression and disrupted function of these proteins can result in abnormal tissue and organ development (Editor: M. G. Mahoney).

“*Desmosomes in developing human epidermis*” by S. Peltonen et al. tackles a rare topic: the distribution of desmosomes and other adhesive structures in the developing epidermis of human embryos. The authors encourage investigators in skin to complement these existing data by mechanistic analyses that can link up with current knowledge obtained from animal models. Of great value for the reader, this paper provides a comprehensive insight into the time line of epidermal morphogenesis from the surface ectoderm to the periderm and stratified epithelium (Editor: E. J. Müller).

##  Disease Models

The paper entitled “*Desmosomal molecules in and out of adhering junctions—normal and diseased states of epidermal, cardiac and mesenchymally derived cells*” by S. Pieperhoff et al. explores the heterogeneity of cell junctions in terms of morphology and composition and how these factors relate to the development of diseases. Special emphasis is given to cell-cell junctions of the mammalian heart, a target organ for severe diseases caused by impaired desmosome function (Editor: P. J. Koch).

“*A new prospective on intercalated disc organization—implications for heart disease*” by J. Li and G. L. Radice reviews arrhythmogenic right ventricular cardiomyopathy/dysplasia (ARVC/D), an inherited fibrotic heart muscle disease resulting from mutations in many desmosomal proteins including plakoglobin, desmoplakin, plakophilin-2, and desmoglein-2. The skin and the heart are two tissues that undergo tremendous mechanical stress. Unlike in the skin, where the adherens junctions are distinct from the desmosomes, in the heart they are integrated into a specialized hybrid structure called the area composita, localized in the cardiac intercalated discs. In their paper, Li and Radice discuss the crosstalk among different junctions and their implications in the pathophysiology of ARVC/D (Editor: M. G. Mahoney).

The paper entitled “*Desmosomal component expression in normal, dysplastic and oral squamous cell carcinoma*” by N. Narayana et al. shows that during malignant transformation, cell-cell adhesion is often reorganized with dramatic changes in various junctional proteins. The authors showed that the desmomal plaque proteins desmoplakin and plakophilin-1 are downregulated in dyplasias and squamous cell carcinomas as compared to control epithelia. The results identify these proteins as potential markers for neoplastic lesions of the oral cavity (Editor: M. G. Mahoney).

In the paper entitled “*Loss of the desmosomal component perp impairs wound healing in vivo*” by V. G. Beaudry et al., the authors investigated cutaneous wound healing in mice with a conditional null mutation in the desmosomal Perp gene. The mice showed a delay in cutaneous wound healing, suggesting that desmosome formation or desmosome remodeling might play an important role during reepithelialization of skin wounds (Editor: P. J. Koch).

Wier et al.'s “*Experimental human cell and tissue models of pemphigus*” discusses the advantages and disadvantages of various in vitro skin models (keratinocyte cultures, raft cultures, reconstructed skin, and human and mouse keratinocyte grafts) used to study the pathophysiology of pemphigus, a group of human autoimmune bullous diseases (Editor: P. J. Koch).

“*Mouse models for blistering skin disorders*” by R. Ganeshan et al. discusses the important roles that autoantibodies play in autoimmune diseases such as pemphigus vulgaris, with antibodies targeting several proteins including the desmosomal cadherins, desmoglein-3, and desmocollin-3. In this paper the authors explore the expression patterns of desmoglein-3 and desmocollin-3 in various stratified epithelial tissues including skin. Furthermore, the authors discuss genetically engineered Dsg3-null and Dsc3-null mice, which develop blistering phenotypes similar to human pemphigus vulgaris patients, thus providing insights into the roles of these proteins in cell-cell adhesion and intra-epidermal blister formation (Editor: M. G. Mahoney).

The paper entitled “*Suprabasal Dsg2 expression limits epidermal blister formation mediated by pemphigus foliaceus antibodies and exfoliative toxins*” by D. Brennan et al. demonstrates that, upregulation of Dsg2 in the upper epidermis of mice can compensate for a loss of Dsg1 function induced by pathogenic pemphigus foliaceus antibodies or exfoliative toxins, thus preventing blistering. This supports the idea that compensatory upregulation of desmogleins could be a therapeutic approach to suppress skin blistering in PF (Editor: P. J. Koch).

“*Apoptotic pathways in pemphigus*” by M. Bektas et al. is a comprehensive review of pemphigus, a group of devastating human autoimmune blistering diseases of the skin and oral mucosa. Although the major uncontested culprits of the disease are the autoantibodies to desmogleins, the pathomechanism of pemphigus is still a heavily debated topic. In this review, they explore the role of cellular apoptosis in pemphigus and summarize substantial evidence suggesting that blister formation can occur independent of apoptosis. However, the authors suggest that the activation of proapoptotic proteins may play important roles in sensitizing the keratinocytes to the acantholytic effects of pemphigus IgG (Editor: M. G. Mahoney).

In “*Targeted immunotherapy with rituximab leads to transient alteration of the IgG autoantibody profile in pemphigus vulgaris*” by Ralf Müller et al., the authors present a clinical evaluation of the B-cell-depleting antibody rituximap in pemphigus patients. The results support the notion that this treatment can lead to a reduction of pathogenic antibodies and, consequently, a temporary disappearance of disease symptoms (Editor, P. J Koch).

In “*Hypothesis concerning a potential involvement of ceramide in apoptosis and acantholysis induced by pemphigus autoantibodies*” by W. B. Bollag, the author presents an exciting and provocative new hypothesis linking certain types of pemphigus to abnormal ceramide metabolism (Editor: P. J. Koch).

*Mỹ G. Mahoney* *Mỹ G. Mahoney* *Eliane J. Müller* *Eliane J. Müller* *Peter J. Koch* *Peter J. Koch*

